# Pharmacist Intervention Program at Different Rent Levels of Geriatric Healthcare

**DOI:** 10.3390/pharmacy5020027

**Published:** 2017-05-19

**Authors:** Conxita Mestres, Anna Agustí, Marta Hernandez, Laura Puerta, Blanca Llagostera

**Affiliations:** 1 School of Health Sciences Blanquerna, University Ramon Llull, Padilla 326, 08025 Barcelona, Spain; 2 Pharmacy Service, HSS Mutuam Girona, Avinguda de França 64, 17007 Girona, Spain; anna.agusti@mutuam.com; 3 Pharmacy Service, EAR Grup Mutuam, Ausias March 39, 08010 Barcelona, Spain; marta.hernandez@mutuam.com (M.H.); bllagostera@mutuam.com (B.L.); 4 Pharmacy Service, HSS Mutuam Güell, Mare de Deu de la Salut 49, 08024 Barcelona, Spain; lpuerta@mutuam.com

**Keywords:** geriatrics, pharmacists’ interventions, inappropriate medication, drug related problems

## Abstract

As a pharmacy service giving pharmaceutical care at different levels of health care for elderly people, we needed a standardization procedure for recording and evaluating pharmacists’ interventions. Our objective was to homogenize pharmacist interventions; to know physicians’ acceptance of our recommendations, as well as the most prevalent drug related problems (DRP); and the impact of the pharmacists’ interventions. To achieve this goal we conducted a one year prospective study at two levels of health care: 176 nursing homes (EAR) (8828 patients) and 2 long-term and subacute care hospitals (HSS) (268 beds). Pharmacists’ interventions were recorded using the American Society of Health-System Pharmacists classification as the basis. Frequency of the different DRP and the level of response and acceptance on the part of physicians was determined. The Medication Appropriateness Index (MAI) was used to evaluate the impact of the interventions on the prescription quality. Patients’ mean age was 84.2 (EAR) and 80.7 (HSS), and in both cases, polypharmacy ≥ 9 drugs was around 63–69%. There were 4073 interventions done in EAR and 2560 in HSS. Level of response: 44% (EAR), 79% (HSS); degree of acceptance of the recommendations: 84% (EAR), 72% (HSS). Most frequent DRP: inappropriate dose, length of therapy, omissions, and financial impact. Drugs for the nervous system are those with the most DRP. MAI values/medication improved from 4.4 to 2.7 (EAR) and 3.8 to 1.7 (HSS). A normalized way of managing pharmacists’ interventions for different health care levels has been established. We are on the way to increasing collaborative work with physicians and we know which DRPs are most prevalent.

## 1. Introduction

Pharmacists have been progressively included in healthcare teams delivering integrated care. The development of pharmaceutical care [1] has permitted pharmacists to provide direct medication-related care with the aim of improving patients’ quality of life. However, even though there have been great advances, they have been accomplished mostly in acute care institutions such as hospitals. These institutions have a larger staff of pharmacists that have been integrated into healthcare teams for a longer time. In other kinds of institutions, like ours, devoted to geriatric patients (long-term care, subacute care, nursing homes), there has been some improvement only recently [2].

Prescriptions for inappropriate drugs for older people are a problem with a high impact on their outcomes, due to their frailty, comorbidities, and polypharmacy. Therefore, drug-related problems (DRP) are more frequent and have a more serious impact.

Adverse drug prevalence among people between 70 and 79 years of age has been found to be 20–30% in contrast to 3–6% for ages 20–29 years old [3]. Moreover, DRP complications result in a higher rate of hospital admissions (6.6–41.3% of older patients) [4]. As a consequence, prescribing potentially unsafe medications may increase pharmacy costs, especially in countries with a higher proportion of older adults in their population [5].

The role of pharmacists in this area has been increasing and—as pointed out by different institutions such as the World Health Organization (WHO) and the American College of Clinical Pharmacy (ACCP)—pharmacists have to be a key member of the healthcare team, due to their privileged position in the review of drug treatments and their knowledge, background, and proximity to healthcare professionals and patients [6,7].

In fact, there have been some examples of the impact of pharmacists on the improvement of patients’ outcomes through their interventions. However, there is not yet a consensus of how to measure these outcomes, and sometimes, results are not conclusive [2,8,9,10]. Our group is specialized in giving healthcare to older people at different levels: nursing homes, long-term care, home care, and subacute areas where the participation of pharmacists in integrative care is usually underdeveloped. Reviewing a document about the implementation of pharmaceutical care issued by the American Society of Hospital Pharmacy at the beginning of the 1990s [11], we found that most of the difficulties in the process are still present in our institutions. Our pharmacy service has been working to incorporate the pharmacist as a member of the care team in order to improve outcomes in our patients, but to achieve it and derive practical improvements in the outcomes of our patients, we have had to address different problems. 

In a first approach, we implemented a guideline to make pharmacologic treatment revisions, and a method for recording our interventions and recommendations to nurses and physicians [12]. After achieving this, we faced other difficulties, such as the homogeneity of the information recorded, which depended on the level of healthcare where our pharmacists were working. Moreover, we wanted to evaluate the impact of our work on the improvement of inappropriate drug prescriptions.

In the present work, we report the process of homogenization of pharmacist interventions done by our pharmacy department, as well as an evaluation of this intervention depending on the type of level of care (HSS or EAR) and the impact on improvements in drug treatment.

## 2. Methods

### 2.1. Setting and Study Population

The project was undertaken at two different levels of health care managed by our institution: two long-term care and subacute care hospitals (HSS): HSS Mutuam Güell (Barcelona, Spain) (165 beds) and HSS Mutuam Girona (103 beds) and 9 teams (EARs) teams composed of a physician and 2 to 3 nurses giving healthcare support to 176 nursing homes (8828 patients) in the city of Barcelona.

The results correspond to all the pharmacist interventions performed between 1 June 2014 and 30 June 2015. All patients in these institutions were eligible to be enrolled, with the only exception of palliative patients in their last days of life.

### 2.2. Intervention

In the two HSS, pharmacists conducted the interventions at different moments of the patient process: admission, during the hospital stay and at discharge. In EAR, due to the small staff of pharmacists, interventions were only performed upon admission to the nursing home.

Information was obtained from different sources such as electronic prescriptions and medical record**s**, as well as the Catalonian Health Care System electronic record (HC3).

Drug-related problems were communicated to the physician through email or telephone and recorded in a database using Microsoft Excel^®^ 2010.

### 2.3. Recording of the Interventions and Outcomes

As one of our main objectives was to standardize the recording of our work, we studied different classification systems [13] of DRP. Previously [12], we used the Pharmaceutical Care Network Europe (PCNE) classification [14], but we found classification difficulties in some of our DRP. We sought a system that included the different problems that we usually find at the different healthcare levels and was easy to use in our daily work (not to increase the workload substantially). We chose the ASHP (American Society Health-System Pharmacists) classification [15] as the most appropriate system for our needs.

### 2.4. Measurement 

Improvement in the appropriateness of drug treatments was evaluated using the MAI (Medication Appropriateness Index) [16]. The significance of the improvement of the MAI value was determined by *p* < 0.0001.

## 3. Results

### 3.1. Demographic Data

In the two HSS, pharmaceutical interventions were done for 1040 patients (48% of the patients admitted to the hospitals during the period of study). In EAR, interventions were performed on 2119 patients (72% of patients admitted to the nursing homes, during the period of study), whose demographic data are shown in Table 1.

In HSSs that treat patients in a more acute situation, there are fewer females and the mean age is lower. In both levels of care, Barthel values denoted a moderate impediment; Pfeiffer index values denoted a higher cognitive deterioration in EAR patients, and thus could be related to the higher mean age. Patients with dementia are mostly found in nursing homes. In both types of institutions there is a high degree of polypharmacy.

### 3.2. Type of Intervention Derived from DRP, Depending on the Institution

Pharmacists performed 4073 interventions in EARs (2.41 interventions/patient) and 2560 in HSS (2.46 interventions/patient). The level of response was 44% in EAR and 79% in HSS. Of the interventions/recommendations for which we obtained an answer, physicians accepted 84% (EAR) and 72% (HSS). Reasons for non-acceptance were reluctance on the part of the physician to change a treatment initiated by a specialist, or being unsure of the consequences of the change recommended by the pharmacist.

DRP that were the origin of the pharmacists’ interventions were classified using the ASHP classification as a basis (Table 2).

In both types of institutions, the most frequent DRP (26.3% and 25.2%) refers to problems related to dosage and the route of administration. In order to study these problems more accurately, we made a subdivision, shown in Table 3. Inappropriate dose is one of the main problems, as it is a frequent prescription error. There are also numerous DRP in relation to the administration frequency in HSS and the excessive length of therapy in EAR. 

In HSS, other important problems are those arising from the financial impact (14.5%), referring to situations where a more cost-effective drug could be used, a condition for which there is no medication prescribed (omissions) with 14.4%, and medications without indication (13.7%). In the first case, this is due to the fact that our patients are taking medications at home that are not included in the hospital formulary; therefore, pharmacists have to propose alternatives based on our interchange guideline. In the case of omissions, the problem usually arises from incomplete information in the clinical record of the patients at the admission. Other frequent problems are duplications, problems with the dose, and the prescription of inappropriate drugs for elderly people.

In EARs, we found 18.2% of cases of patients with medications without indication (18.2%) due to an incomplete record at admission. Omissions are also frequent (13.7%), and inappropriate drugs in geriatrics (11.9%) are also important. Problems arising from the financial impact of therapy are high (10%), as nursing homes attended by our EAR team belong to different private purveyors; and we do not have a defined formulary, but our pharmacists recommend changes for more cost-effective alternatives. 

ATC most implicated in DRP in both institutions is N (nervous system) (Table 4). In EAR, it is followed by B (blood) and A (alimentary tract). In HSS, there is more dispersion with values > 10% in groups such as A, C (cardiovascular), B, and J (anti-infectives).

#### 3.2.1. Level of Acceptance

If we assess the degree of acceptance of the interventions, taking into account the 10 most frequent causes of the intervention (Table 5 and Table 6), they range between 59.5 and 35.9% HSS, and between 57.9 and 28.5% in nursing homes. 

The mean MAI values per medication, after implementation by physicians of a pharmacist’s recommendations in EARs decreased from 4.4 to 2.7 (*p* < 0.0001); in HSS, the values decreased from 3.8 to 1.7 (*p* < 0.0001) (Table 7).

## 4. Discussion

With this project, we have been able to find a suitable classification for documenting and recording the interventions/recommendations made by our pharmacists in different types of institutions giving care to elderly people. Thanks to this, now we can review and study some of the differences found in the DRP detected as well as other problems to make improvements.

We have seen differences in levels of response by physicians between HSS and EAR. In the latter, the response is somewhat low (44%) compared to that of HSS, which was 79%. We have discussed this point with our physicians, and they state that the lack of feedback is due to workload, which makes it difficult for them to contact the pharmacist to discuss all DRPs. Curiously, we have noted that there are occasions where they have made the changes recommended by our pharmacists in the patient treatment, but they fail to communicate it to us. Other reasons for this low level of response, compared with HSS, may be that although we have been working in this area with physicians for some time, they still have not adapted this to their day-to-day the interaction with the pharmacist. Moreover, in HSS, we are working in the same building with similar schedules, making communication easier.

Our strategy has been to increase meetings with physicians and report feedback of the problems detected; this continuing information has made them more receptive and understanding of the importance of taking our recommendations into account and giving us information about what they plan to do; this is an approach that has already been described as effective in the acceptance rates [17,18]. As a consequence, we have observed that since we started the program, the level of response and acceptance is increasing (in the last semester of 2015 we had a 60% of response rate).

As for the type of DRP found, we would like to highlight that we have a high degree of problems related to dosage, schedule, and length of therapy, which we are now reviewing with physicians. Incomplete information in the records at admission (medication without a related diagnosis or indication for a drug that is not prescribed) is also to be considered. We also want to point out that a small percentage of drug omissions were detected, after informing the physician in charge. We have found these justified, because they were medications that the physicians found relatively unnecessary or not appropriate during hospitalization. 

The prescription of inappropriate drugs for older people is higher in EAR than HSS. We think that a possible explanation is that in HSS we work with a formulary adjusted to the needs of our geriatric population, and in recent years, we have worked extensively with our physicians [19] on this problem. Therefore, we think that the development of formularies and interchange programs at admission in nursing homes is necessary.

In HSS, the therapeutic interchange is accepted in 59.5%; thus, we think that there is still room for a wider range of improvement. Physicians continue to be especially reluctant to change medications that patients are taking at home or that have been prescribed by specialists. In reference to the ATC group to which the drugs with more DRP belong, we have found that in all cases, the high percentage is for nervous system drugs. This is not surprising, taking into account that this is the group with more drugs prescribed, and includes drugs for dementia, analgesics, and psychoactive drugs. In HSS, we found that there are more ATC groups with high incidence of problems; for example, we have values of 10.1% for anti-infectives, versus only 1.5% in EARs. This can be explained by anti-infectives having a higher degree of prescriptions in HHS due to the fact that the patients are in a more acute situation with their illness, and infectious processes in many cases are the cause of admission. Other ATC groups with a high prevalence of problems are those related to the alimentary tract (A), especially antidiabetics and proton bomb inhibitors; cardiovascular (C), for which we have a high degree of therapeutic interchange in antihipertensives; and problems with anticoagulants (B).

On the whole, we also consider it to be very important that many of the interventions done are derived from safety problems; therefore, the work of the pharmacists increases the safety of the treatment in many ways, such as reducing the use of inappropriate drugs or detecting prescription errors.

Even though evaluating the impact of these interventions is quite complicated, we found that MAI scores per drug improved when recommendations of pharmacists were implemented by physicians. In EAR, the mean MAI value before intervention was of 4.4, and at HSS it was lower: 3.7. These values and improvement**s** obtained (2.7 in EAR and 1.7 in HSS) are similar to those found by other authors, working with a similar population [20].

The use of the MAI score to evaluate improvements in prescriptions has many limitations. For example, it does not take into account the improvements derived from the correction of drug omissions. However, in a revision done by their designers after 20 years of use, they pointed out different studies relating high MAI scores with unscheduled ambulatory or emergency department visits, inadequate blood pressure control, or adverse drug events [21]. Taking all this into account, our main goals in the future are—besides improving physicians’ response to our work—to find and implement other methods and means to evaluate the impact of pharmacists’ interventions, including an economic evaluation.

## 5. Conclusions

- The standardization of recording pharmacists’ interventions improves the management of drug related problems.
- There is still a long way to go to incorporate the pharmacist in day-to-day work with physicians in nursing homes.
- Prescriptions for non-appropriate medication for older people continue to be a recurrent DRP.
- The implication of pharmacists in medication reviews leads to a quality improvement in the prescriptions.
- It is important to give information and training to all health care professionals concerning the benefits of collaborative work with pharmacists.
- Guidelines, formularies, and interchange programs should be implemented in nursing homes.
- Knowing the most prevalent and serious DRPs allows us to focus on their prevention.

## Figures and Tables

**Table 1:** Demographic data of the patients.

Institution	Patients (No.)	Female (%)	Mean Age	Polypharmacy ≥ 9 Drugs	Barthel Index	Pfeiffer Index
EAR	2119	70.2	84.2 ± 7.8	69.0%	49	6
HSS	1040	58.9	80.7 ± 9.9	63.4%	45	3

EAR: Teams attending nursing homes; HSS: long-term care and subacute care hospitals. Barthel index scores: 0–20: totally dependent, 20–35: severe dependence, 40–55: moderate dependence, 60–95: mild dependence, 100: independent. Pfeiffer index score: 0–2: normal, 3–4: mild cognitive deterioration, 5–7: moderate cognitive deterioration, 8–10: significant cognitive deterioration.

**Table 2:** Interventions performed for the different DRP found, based on the ASHP classification.

Drug Related Problem	HSS		EAR	
	*n* = 2560		*n* = 4073	
1—Medication with no indication	353	13.7%	741	18.2%
2—Condition for which no drug is prescribed	369	14.4%	558	13.7%
3—Medication prescribed inappropriately for a particular condition	206	8.0%	485	11.9%
4—Inappropriate dose, dosage form, schedule, route of administration, or method of administration	662	26.3%	1025	25.2%
5—Therapeutic duplication	224	8.8%	174	4.3%
6—Prescribing of medication to which the patient is allergic	1	0.1%	22	0.5%
7—Actual and potential adverse drug events	59	2.3%	116	2.9%
8—Actual and potential drug-drug, drug-disease, drug-nutrient, and drug-laboratory test interactions that are clinically significant	109	4.3%	149	3.7%
9—Interference with medical therapy by social or recreational drug use	0	0.0%	0	0.0%
10—Failure to receive the full benefit of prescribed therapy	85	3.3%	220	5.4%
11—Problems arising from the financial impact of therapy	370	14.5%	409	10.0%
12—Lack of understanding of the medication	101	4.0%	72	1.8%
13—Failure of the patient to adhere to the regimen	11	0.4%	5	0.1%

EAR: Teams attending nursing homes; HSS: long-term care and subacute care hospitals.

**Table 3:** Different types of DRP in Category 4.

Drug Related Problem	HSS		EAR	
4.1—Inappropriate dose	208	8.1%	214	5.2%
4.2—Inappropriate dose, renal insufficiency	15	0.6%	97	2.4%
4.3—Inappropriate dose, hepatic insufficiency	0	0.0%	33	0.8%
4.4—Dosage form	38	1.5%	4	0.1%
4.5—Schedule	187	7.3%	55	1.3%
4.6—Length	101	4.0%	675	16.6%
4.7—Route of administration	101	4.0%	44	1.1%
4.8—Method of administration	12	0.5%	0	0.0%

EAR: Teams attending nursing homes; HSS: long-term care and subacute care hospitals.

**Table 4:** ATC group of the drugs mots implicated in DRP in EARs and HSS.

ATC Group	EARs	HSS
A	13.7%	16.1%
B	17.7%	10.9%
C	9.3%	15.8%
D	0.8%	0.1%
G	4.7%	2.9%
H	1.3%	2.8%
J	1.5%	10.1%
L	0.7%	1.1%
M	5.0%	4.0%
N	41.5%	25.5%
R	2.9%	6.3%
S	1.0%	1.7%
V	0.1%	2.1%

EAR: Teams attending nursing homes; HSS: long-term care and subacute care hospitals.

**Table 5:** Acceptance degree of the most frequent DRP in HSSs.

HSS	Acceptance Degree (%)
11—Problems are arising from the financial impact of therapy	59.5
2—Condition for which no drug is prescribed	66.4
1—Medication with no indication	62.9
5—Therapeutic duplication	55.4
4.1—Inappropriate dose	65.9
3—Medication prescribed inappropriately for a particular condition	35.9
4.5—Schedule	44.4
4.6—Length	57.4
4.7—Route of administration	59.4

**Table 6:** Acceptance degree of the most frequent DRP in EARs.

EAR	Acceptance Degree (%)
1—Medication with no indication	44.3
4.6—Length	43.1
2—Condition for which no drug is prescribed	34.6
3—Medication prescribed inappropriately for a particular condition	57.9
11—Problems are arising from the financial impact of therapy	37.2
10—Failure to receive the full benefit of prescribed therapy	36.8
4.1—Inappropriate dose	28.5
5—Therapeutic duplication	36.8
7—Actual and potential adverse drug events	36.2

**Table 7:** Mean MAI values per medication

Institution	Pre-Intervention	Post-Intervention	Min	Max	*p* Value
EAR	4.4	2.7	0	17	*p* < 0.0001
HSS	3.8	1.7	0	15	*p* < 0.0001

EAR: Teams attending nursing homes; HSS: long-term care and subacute care hospitals.
